# Simultaneous Generation of Multiple Three-Dimensional Tractor Curve Beams

**DOI:** 10.1186/s11671-019-2895-0

**Published:** 2019-03-05

**Authors:** Jun Wu, Xinquan Tang, Jun Xia

**Affiliations:** Joint International Research Laboratory of Information Display and Visualization, School of Electronic Science and Engineering, Southeast University, Nanjing, 210096 China

**Keywords:** Beam shaping, 3D tractor beam, Computer holography

## Abstract

A tractor beam, which has the ability to attract objects, is a class of special optical beams. Currently, people are using the holographic technology to shape complex optical tractor beams for both fundamental research and practical applications. However, most of the work reported is focusing on generating two-dimensional (2D) tractor beams and simple three-dimensional (3D) tractor beams, which has limitations in the further development on mechanism and application of beam shaping. In the present work, we are introducing our study in designing multiple 3D tractor beams with spatial location regulated independently. Meanwhile, each individual beam could be prescribed along arbitrary geometric curve and twisted at arbitrary angles as desired. In our method, the computer-generated hologram (CGH) of each curve is calculated, and all the CGHs are multiplexed and encoded into one phase-only hologram by adding respective linear phase grating such that different 3D curves appeared in the different positions of the focal regions. We experimentally prove that the generation of optical tractor beams at 3D configuration can be readily achieved. The generated beams in the present study are especially useful for applications such as multiple micro-machining optical trapping and complex 3D manipulation.

## Introduction

Long ago, people have demonstrated the ability of light to exert forces. The idea of attracting objects with optical beams has also arrested our attention for a long time. Due to the phase singularity and unique orbital angular momentum, optical vortex has important research value in the fields of optical micromanipulation, quantum communication, optical imaging, and optical measurement [1–6]. Although the technology for generating optical vortices has been developed and may be valuable in various applications, the efficiency of a single optical vortex is still low. In order to capture multiple particles at the same time and operate different particles separately, the generation of optical vortex arrays has become a hot topic [7, 8].

Recent theoretical studies [9–13] have shown that a tractor beam is a traveling wave that can transport illuminated material along its length back to its source. New advances in laser beam control have led to the experimental realization of tractor beams [14, 15]. One important type of 3D vortex trap is the so-called solenoid beam that exhibits a fixed spiral shape around the optical axis [16], in which the phase gradient can be prescribed along this curve to obtain a tractor beam. It was achieved by imposing helical phases to a collinear superposition of Bessel beams. Ruffner and Grier [17] experimentally demonstrated and analyzed the properties of a class of tractor beam obtained by the interference of two coaxial Bessel Beams that differ in their axial wave numbers. In 2013, Rodrigo et al. present a method for efficient generation of tractor beams by loading designed phase-only holograms into the spatial light modulator (SLM) and meanwhile irradiating the SLM with lasers. They used the technique to allow the generation of high-intensity gradient (HIG) beams whose phase and intensity are prescribed based on the computer-generated hologram (CGH) [18]. They experimentally proved that the beams in distinct 3D geometries could be shaped. The HIGs and phase gradient forces are crucial for the construction of 3D laser traps that are able to move multiple particles even against light radiation pressure [19]. Rodrigo also showed that a freestyle laser trap, including HIG and phase gradient forces, was able to confine multiple particles and drive their motion [20]. However, most of the work reported is focusing on generating simple 3D tractor beams, which has limitations in the further development of applications of beam shaping. Based on the above analysis, advanced beam-shaping technology for the generation of multiple 3D tractor beams is urgently needed.

In this paper, we demonstrate the method for generation of multiple 3D tractor beams using the modified holographic beam shaping technique, where all the CGHs are multiplexed and encoded into one phase-only hologram by adding respective linear phase grating. We design multiple 3D tractor beams that are twisted at different angles. Such >new tractor beams are expected to expand the application field of optical vortices and are potentially useful in the realization of super-performance multiple optical applications.

## Methods

**Fig. 1:**
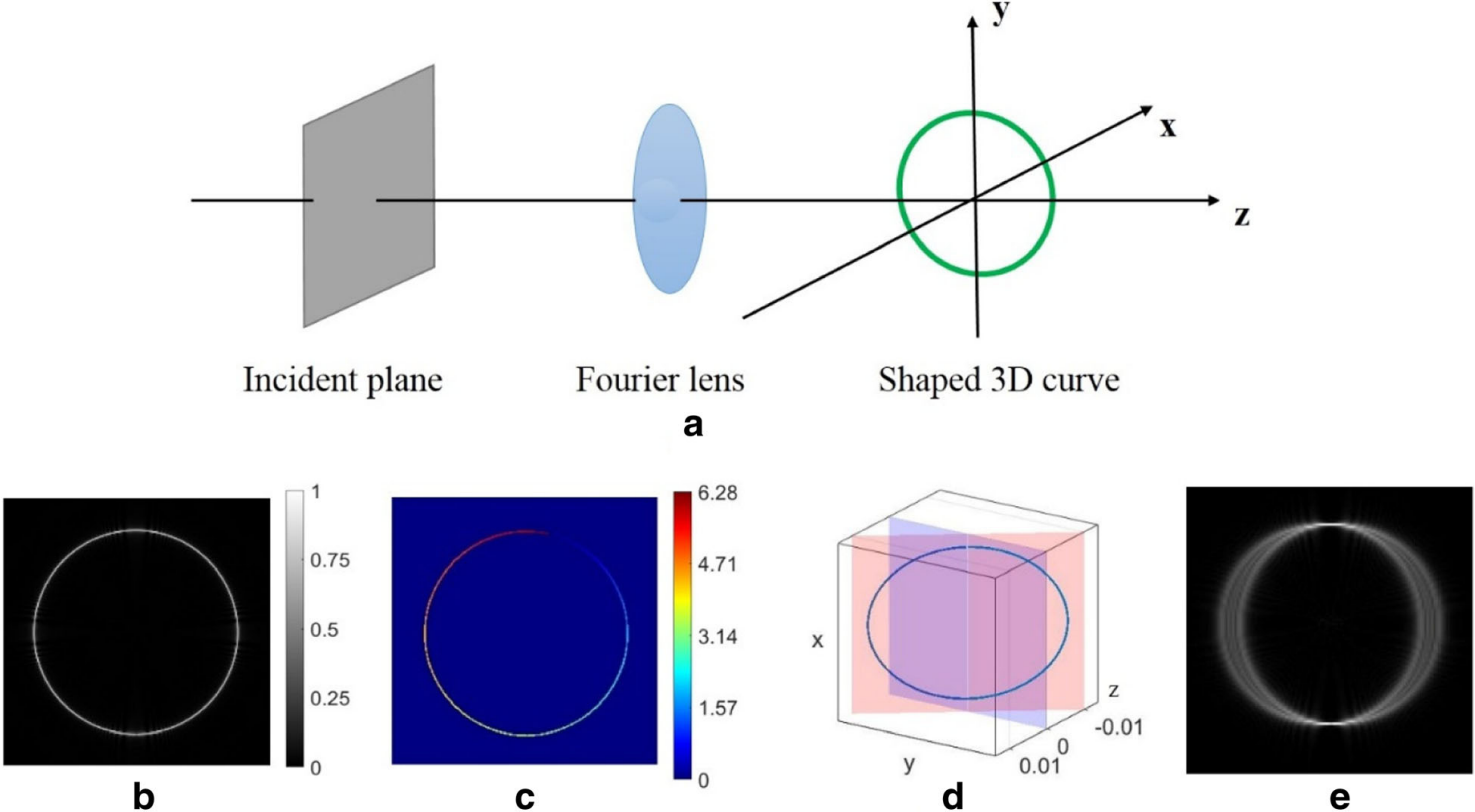
**a** Scheme of holographic 3D beam shaping technique. **b**, **c** Reconstructed intensity and phase distribution of the 2D ring curve at the focal plane. **d** Spatial schematic of a ring curve tilted relative to the plane *z* = 0. **e** The ring tractor beam focused on the focal plane (*z* = 0)

Figure 1a shows the scheme of a holographic 3D beam shaping technique in [18] that allows designing complex beams whose intensity and phase distribution follow a prescribed 3D curve. Encoding the complex amplitude field into phase holographic gratings is a method to calculate CGH. Specifically, in order to generate a desired focal beam, the complex amplitude of the incident plane is given by the expression:

1 $$ G\left(x,y\right)={\int}_0^{2\pi}\varphi \left(x,y,t\right)\phi \left(x,y,t\right)\sqrt{{\left[{x_0}^{\hbox{'}}(t)\right]}^2+{\left[{y_0}^{\hbox{'}}(t)\right]}^2} dt $$

The terms *ψ*(*x*, *y*, *t*) and *φ*(*x*, *y*, *t*) in Eq. (1) are determined by

2 $$ \varphi \left(x,y,t\right)=\exp \left( i\pi {z}_0(t)\raisebox{1ex}{${\left[x-{x}_0(t)\right]}^2+{\left[y-{y}_0(t)\right]}^2$}\!\left/ \!\raisebox{-1ex}{$\lambda {f_0}^2$}\right.\right) $$

3 $$ \phi \left(x,y,t\right)=\exp \left(\frac{i}{\omega_0^2}\left[{yx}_0(t)-{xy}_0(t)\right]+\frac{i\sigma}{\omega_0^2}{\int}_0^t\left[{x}_0\left(\tau \right){y}_0^{\hbox{'}}\left(\tau \right)-{y}_0\left(\tau \right){x}_0^{\hbox{'}}\left(\tau \right)\right] d\tau \right) $$

[*x*_0_(*t*), *y*_0_(*t*), *z*_0_(*t*)] represents the prescribed 3D curve in the Cartesian coordinate with *t*∈[0,2*π*]. *f*_0_ and *λ* refer to the focal length of the Fourier lens and the wavelength, respectively.

Eq. (1) allows calculating the incident complex field (namely, complex CGH) that can shape a structurally stable focal beam with special intensity distribution and phase gradient (helical phase along the curve). We first consider a 2D ring curve *x*_0_(*t*) = Rcos(*t*), *y*_0_(*t*) = Rsin(*t*). The intensity distribution of the resulting beam is displayed in Fig. 1b. The phase distribution of the ring is well defined along curves under the topological charge of m = 1 [see Fig. 1c]. We consider a tilted ring in Fig. 1d. The plane of the ring curve inclined to a certain angle on the basis of the plane *z* = 0. In this case, the beam is focused appearing on the top and bottom points [seen in Fig. 1e].

In order to multiplex various tractor curve beams partially separated in the focal field, each complex CGH calculated by Eq. (1) must be encoded with a unique carrier frequency. This can be achieved by adding a linear phase grating to the hologram of each beam. Linear gratings in combination with spatial filters are commonly used to isolate the first diffraction order from undesired zero and higher diffraction orders. The transfer function of a linear phase grating is given as

4 $$ {\varphi}_i\left(x,y\right)={kz}_i\sqrt{1-\raisebox{1ex}{${x}^2$}\!\left/ \!\raisebox{-1ex}{${f_0}^2$}\right.-\raisebox{1ex}{${y}^2$}\!\left/ \!\raisebox{-1ex}{${f_0}^2$}\right.}+k\left(\raisebox{1ex}{${xu}_i$}\!\left/ \!\raisebox{-1ex}{${f}_0$}\right.+\raisebox{1ex}{${yv}_i$}\!\left/ \!\raisebox{-1ex}{${f}_0$}\right.\right) $$

*u*_*i*_ and *v*_*i*_ are the spatial coordinates of the generated beam in the far field, achieved with a Fourier lens of focal length *f*_0_. *k* = 2*π*/*λ* is the wave number, and *z*_*i*_ is the axial shifted displacement away from the focal plane (Fourier plane). In order to generate tractor curve beams simultaneously, the expressions of the final complex CGH need to be added together by

5 $$ H\left(x,y\right)=\sum \limits_{i=1}^n{G}_i\left(x,y\right)\cdotp \exp \left[i{\varphi}_j\left(x,y\right)\right] $$

## Results and Discussion

**Fig. 2:**
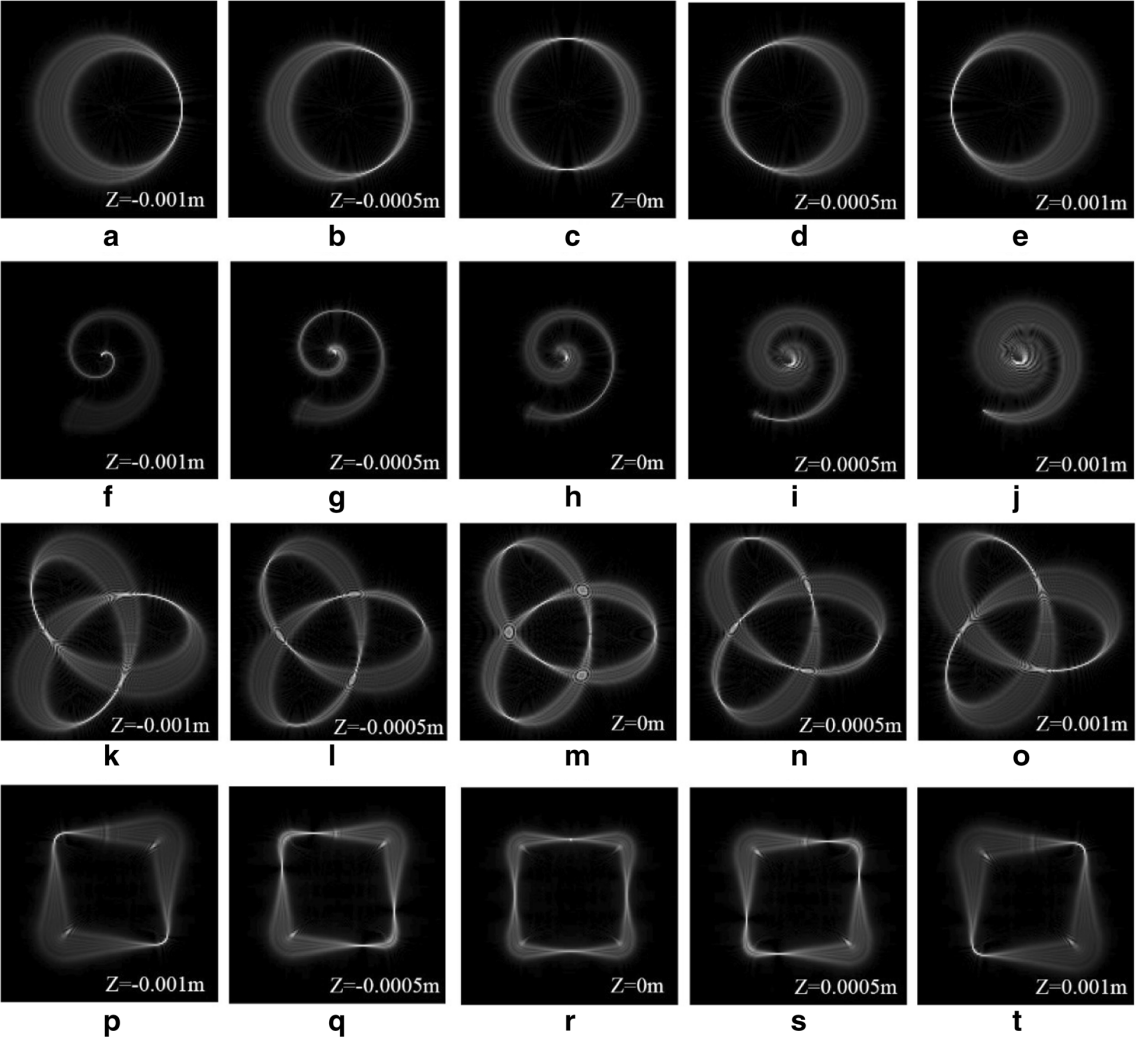
Simulation results of 3D tractor beams**. a**–**e** Ring curve of 3D tractor beams focused on different *z* planes. **f**–**j** Archimedean spiral of 3D tractor beams focused on different *z* planes. **k**–**o** Trefoil-knotted curve of 3D tractor beams focused on different *z* planes. **p**–**t** Square curve of 3D tractor beams focused on different *z* planes

Light field regulation at 3D configuration is very meaningful in practical applications, such as 3D manipulation of particles in a fluid environment. Therefore, we study the generation of HIG beams whose intensity and phase are prescribed along 3D curves of different shapes. Specifically, we consider a tilted ring Fig. 2a–e, an Archimedean spiral Fig. 2f–j, a trefoil-knotted curve Fig. 2k–o, and a square curve Fig. 2p–t. The corresponding curve expressions are provided in Table 1. These 3D structures are revealed along the beam propagation in the focal region. The beam intensity distributions measured in the focal plane (*z* = 0) are shown in the third column of Fig. 2. The *Z* coordinates corresponding to other columns of Fig. 2 are marked in the simulation diagrams.

**Table 1:** Table 1

Type of curve	*x*0(*t*)	*y*0(*t*)
Ring curve	Rcos(*t*)	Rsin(*t*)
Archimedean spiral	− Rtcos(10t)	− Rtsin(10*t*)
Trefoil-knotted curve	Rcos(*t*) − 2Rcos(2*t*)	Rsin(*t*) + 2Rsin(2*t*)
Square curve	− 2Rcos(*t*) + 0.3Rcos(*kt*)	− 2Rsin(*t*) + 0.3Rsin(*kt*)

**Fig. 3:**
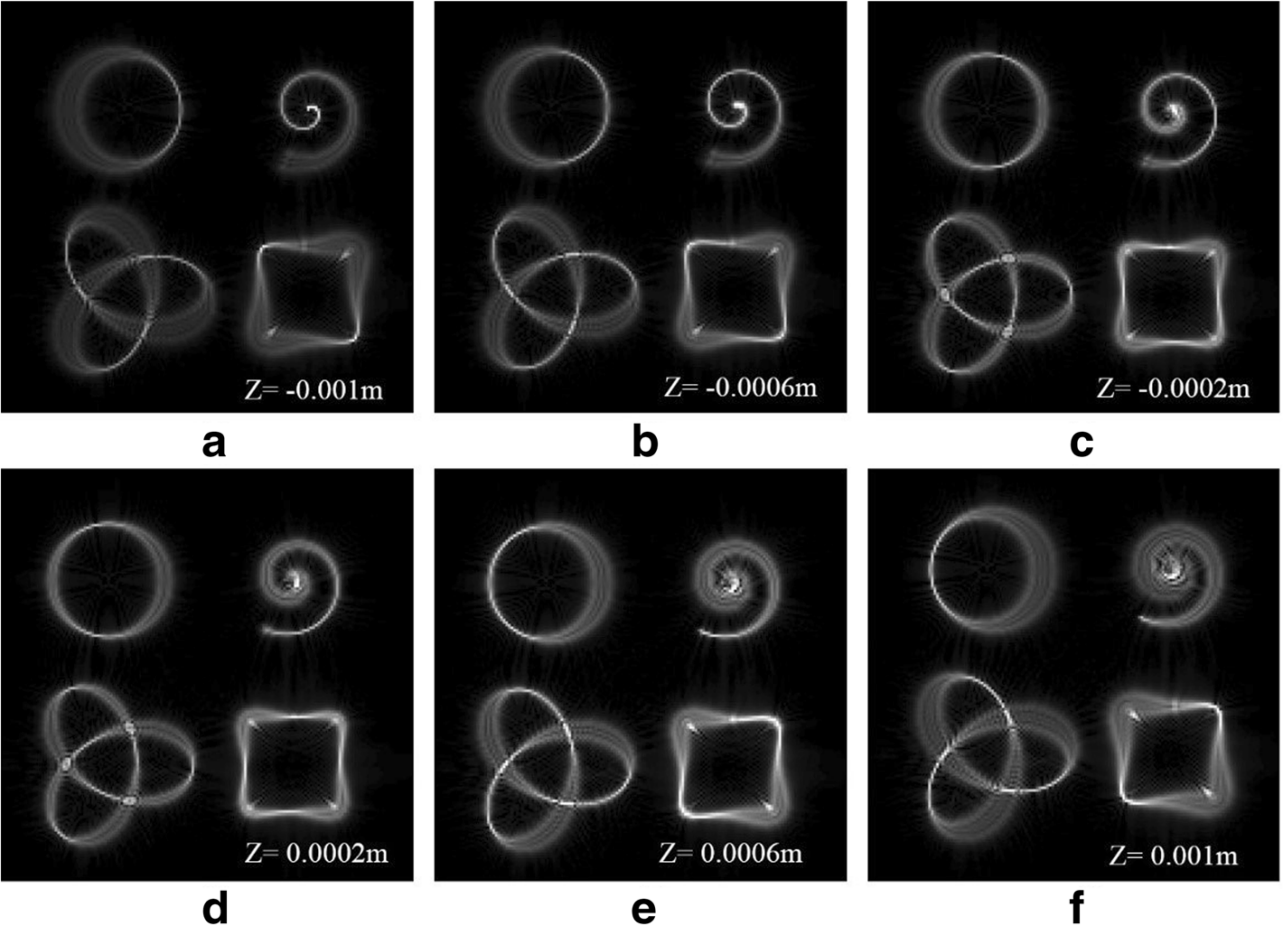
Simulation results of multiple 3D tractor beams at different locations. **a**–**c** Reconstructed intensity of the beams before the focal plane. **d**–**f** Reconstructed intensity of the beams after the focal plane

In optical micromanipulation fields, tractor beams with different degrees of 3D distortion can play a greater role in applications. However, for higher efficiency, it is highly desired to synchronously carry out differentiated manipulations at different locations. Therefore, we design multiple tractor beams of four patterns simultaneously, each of which may be used to confine given particles in any prescribed geometric curve with a certain degree of 3D distortion. The relative position of the tractor beams can be designed. In order to show that multiple tractor beams are focused on the 3D region, we have selected six 2D planes to observe. The tractor beams are focused on different 2D planes, seen in Fig. 3. These 3D structures are revealed along the beam propagation in the focal region.

**Fig. 4:**
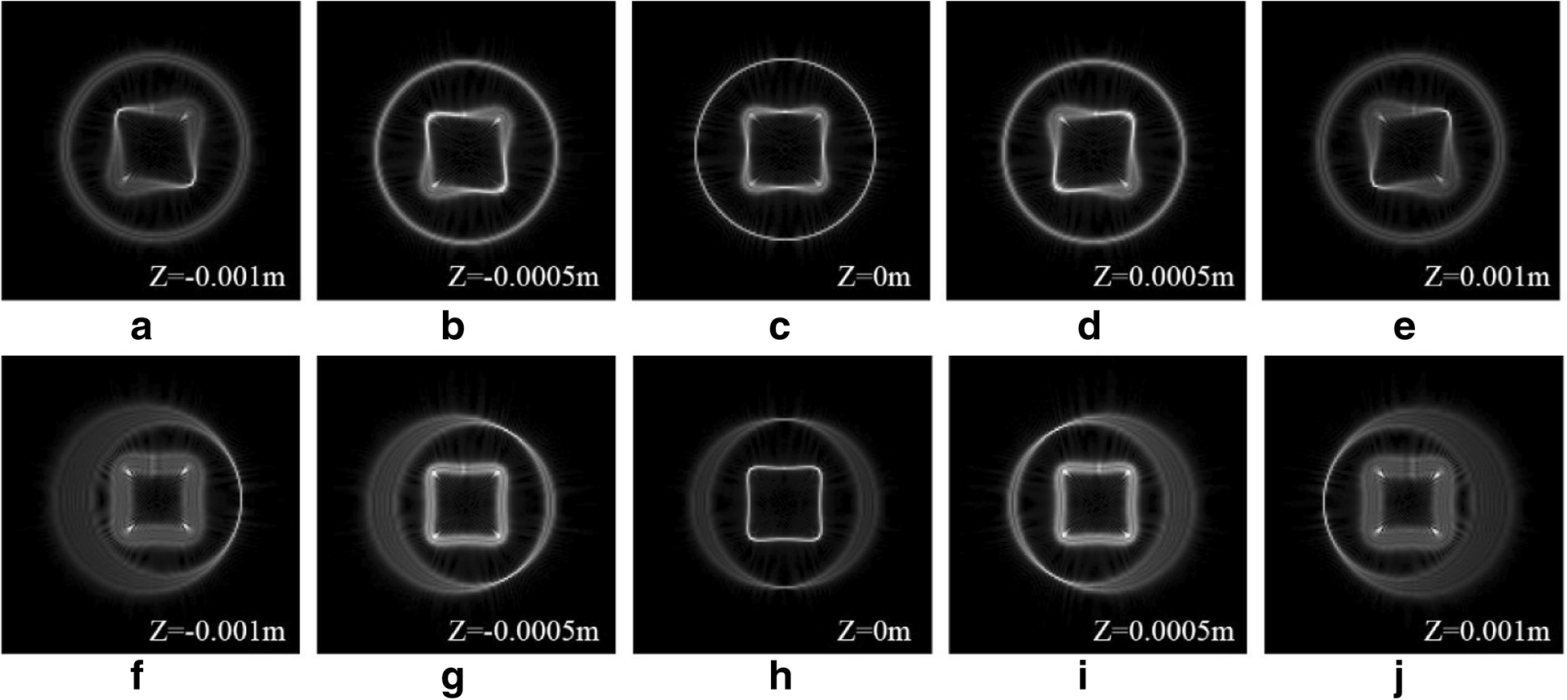
Simulation results of copper-like nested graphic tractor beams. **a**–**e** Beams shaped in a tilted square curve are focused on different *z* planes. **f**–**j** Beams shaped in a tilted ring curve are focused on different *z* planes

In order to observe the interaction of multiple manipulated particles, we designed copper-like nested graphic tractor beams. The 3D distortion and shapes of the inner and outer beams can be separately designed. The ring curve is focused on the plane (*z* = 0), and the square curve has a certain degree of 3D distortion [seen in Fig. 4a–e]. The square curve is focused on the plane (*z* = 0), and the ring curve has a certain degree of 3D distortion [seen in Fig. 4f–j]. The beam intensity distributions measured in the focal plane (*z* = 0) are shown in the third column of Fig. 4. The *z* coordinates corresponding to other columns of Fig. 4 are marked in the simulation diagrams. The shape of the tractor beams can be flexibly adjusted to control the particles at different positions.

**Fig. 5:**
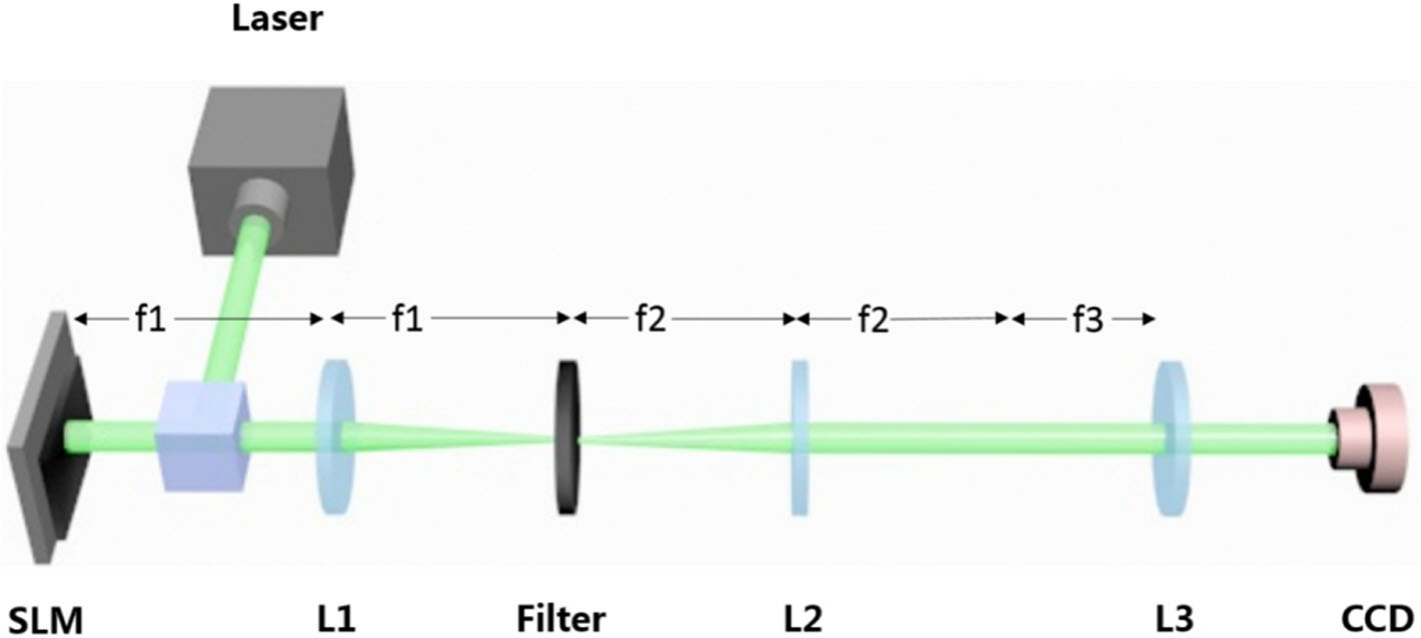
Experimental setup. The hologram is addressed into the SLM, which is illuminated by a collimated laser beam. After the beam passes through lens 1, the desired pattern can be filtered with diaphragm. Then resulted beams pass through lens 2 and lens 3 and can be captured by the camera

**Fig. 6:**
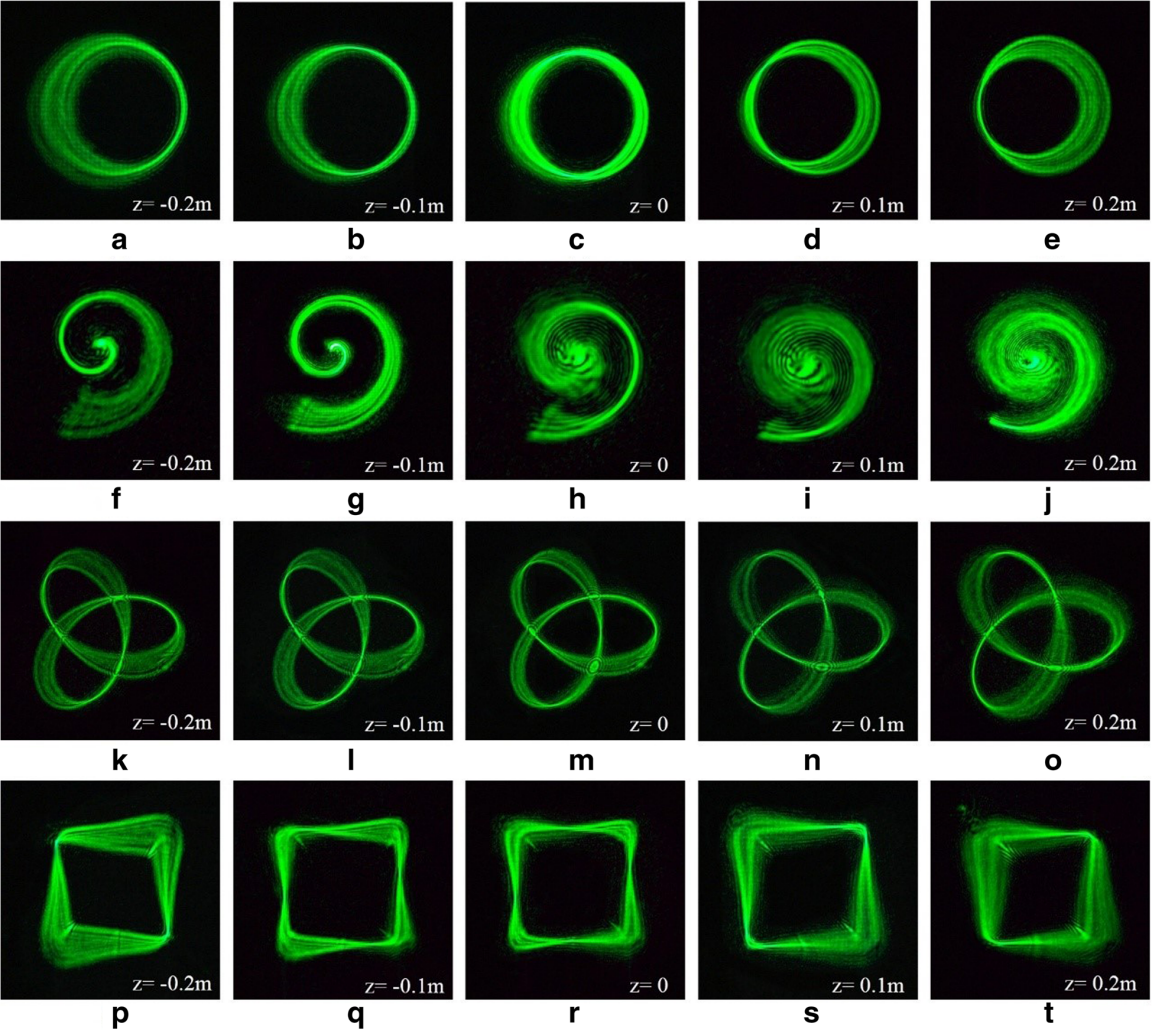
Experimental results of 3D tractor beams. **a**–**e** Ring curve of 3D tractor beams focused on different *z* planes. **f**–**j** Archimedean spiral of 3D tractor beams focused on different *z* planes. **k**–**o** Trefoil-knotted curve of 3D tractor beams focused on different *z* planes. **p**–**t** Square curve of 3D tractor beams focused on different *z* planes

Optical experiments have been performed to verify that the method introduced above can be used to achieve the purpose of focusing multiple 3D tractor beams in tunable focusing regions. As shown in Fig. 5, the optical setup for generating the 3D tractor beam is composed of a liquid crystal spatial light modulator (SLM), a 4f filtering system, and a Fourier transform (focusing) lens. A solid-state laser with a wavelength of 532 nm is collimated to plane wave illumination. The SLM (Holoeye Pluto, 8 pixel pitch, 1920 × 1080 resolution) is utilized to address a phase-only CGH. We use the double-phase method [18, 20] to encode the complex CGH *H*(*x*,*y*) calculated by Eq. (4) into a phase-only CGH. It consists in the encoding of the complex function as a hologram into the SLM. The beam modulated by SLM is then projected to the back-aperture of the Fourier transform lens (*f* = 400 mm) through a 4f optical filtering configuration. A charge-coupled device (CCD) camera is placed at the Fourier plane of the focusing lens to record the generated intensity patterns. The results of the 3D tractor beams are shown in Fig. 6. Although the resulting beams have errors after passing through the 4f optical system, they are in good agreement with the simulation results.

**Fig. 7:**
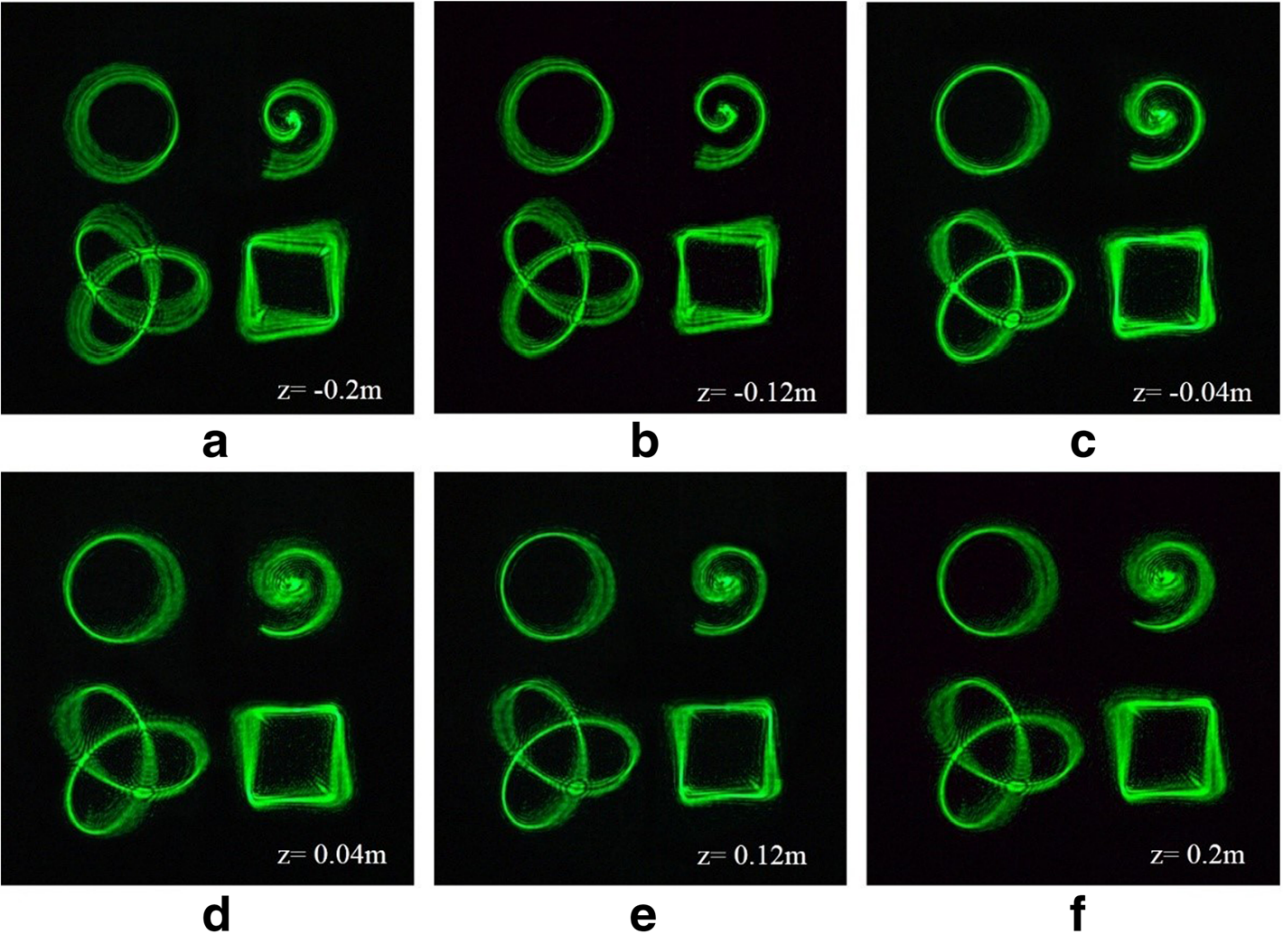
Experimental results of multiple 3D tractor beams at different locations. **a**–**c** Reconstructed intensity of the beams before the focal plane. **d**–**f** Reconstructed intensity of the beams after the focal plane

The results of the multiple 3D tractor beams are shown in Fig. 7. We have selected six 2D planes to observe, which is convenient for comparison with simulation. The simulation results are in good agreement with the experimental results. It is verified that this method can generate multiple 3D tractor beams flexibly and efficiently. Different beams with a certain degree of 3D distortion can confine particles.

**Fig. 8:**
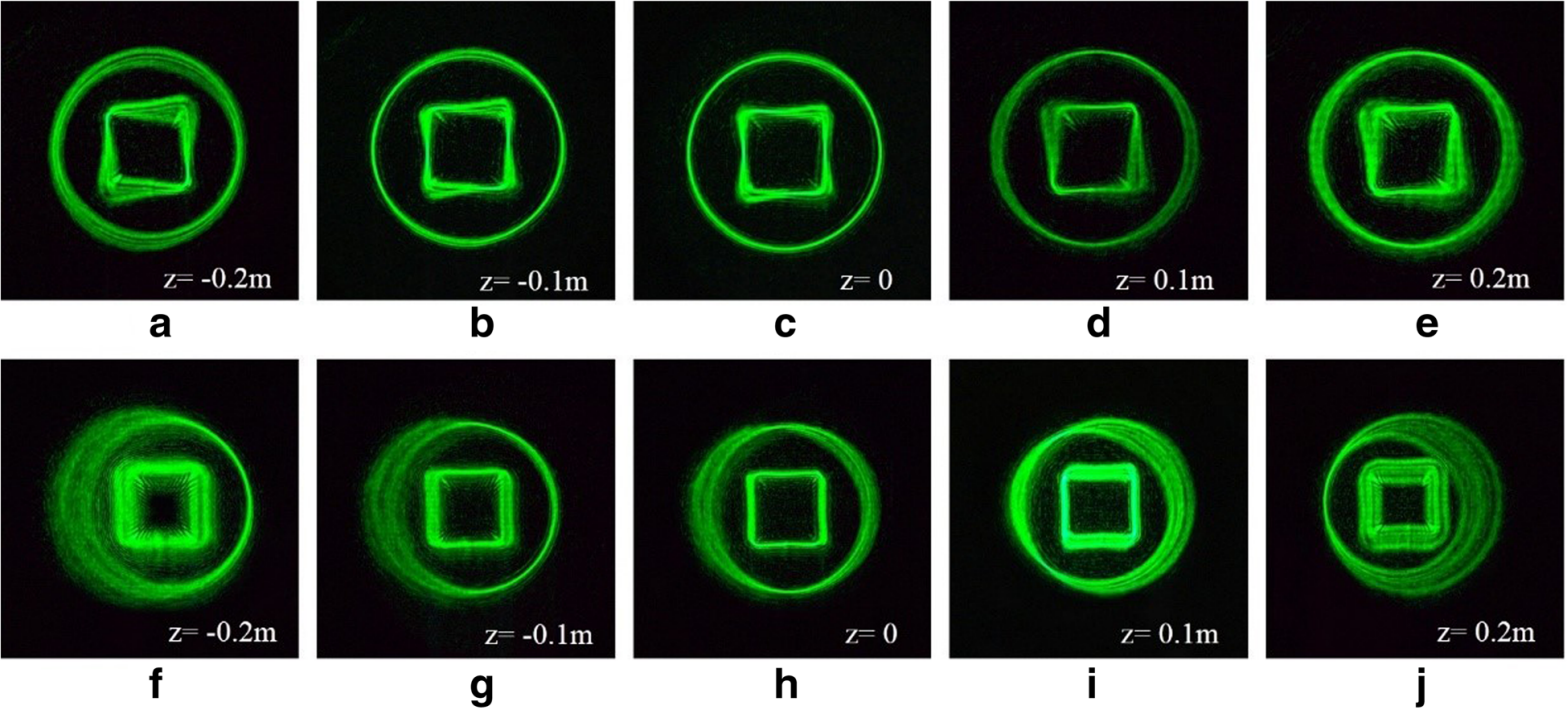
Experimental results of copper-like nested graphic tractor beams. **a**–**e** Beams shaped in a tilted square curve are focused on different *z* planes. **f**–**j** Beams shaped in a tilted ring curve are focused on different *z* planes

 The results of copper-like nested graphic tractor beams are shown in the Fig. 8. The simulation results are in good agreement with the experimental results. Thus, the two nested beams hardly interact with each other. The tractor beams can be used for multi-particle manipulation on different curves.

## Conclusion

We design multiple 3D tractor beams with spatial location regulated independently. Meanwhile, each individual beam could be prescribed along arbitrary geometric curves and twisted at arbitrary angles as desired. We theoretically and experimentally prove that the generation of optical tractor beams at 3D configuration can be readily achieved. High-intensity gradients and phase gradients have the ability to capture particles. At present, experiments have been carried out and the optical vortex beams damage the particles to a minimum extent. Our work broadens the types of tractor beams. It is believed meaningful and useful for further development of tractor beams for multiple optical applications.

## Abbreviations

2D: Two-dimensional
3D: Three-dimensional
CGH: Computer-generated hologram
HIG: High intensity gradient
SLM: Spatial light modulator
